# Mucosal IgA Prevents Commensal *Candida albicans* Dysbiosis in the Oral Cavity

**DOI:** 10.3389/fimmu.2020.555363

**Published:** 2020-10-22

**Authors:** Nicolas Millet, Norma V. Solis, Marc Swidergall

**Affiliations:** ^1^Division of Infectious Diseases, Harbor-UCLA Medical Center, Torrance, CA, United States; ^2^Institute for Infection and Immunity, The Lundquist Institute at Harbor-UCLA Medical Center, Torrance, CA, United States; ^3^David Geffen School of Medicine at UCLA, Los Angeles, CA, United States

**Keywords:** oropharyngeal candidiasis, commensalism, fungi, host-pathogen interaction, B cell, antifungal immunity

## Abstract

The fungus *Candida albicans* colonizes the oral mucosal surface of 30–70% of healthy individuals. Due to local or systemic immunosuppression, this commensal fungus is able to proliferate resulting in oral disease, called oropharyngeal candidiasis (OPC). However, in healthy individuals *C. albicans* causes no harm. Unlike humans mice do not host *C. albicans* in their mycobiome. Thus, oral fungal challenge generates an acute immune response in a naive host. Therefore, we utilized *C. albicans* clinical isolates which are able to persist in the oral cavity without causing disease to analyze adaptive responses to oral fungal commensalism. We performed RNA sequencing to determine the transcriptional host response landscape during *C. albicans* colonization. Pathway analysis revealed an upregulation of adaptive host responses due to *C. albicans* oral persistence, including the upregulation of the immune network for IgA production. Fungal colonization increased cross-specific IgA levels in the saliva and the tongue, and IgA^+^ cells migrated to foci of fungal colonization. Binding of IgA prevented fungal epithelial adhesion and invasion resulting in a dampened proinflammatory epithelial response. Besides CD19^+^ CD138^−^ B cells, plasmablasts, and plasma cells were enriched in the tongue of mice colonized with *C. albicans* suggesting a potential role of B lymphocytes during oral fungal colonization. B cell deficiency increased the oral fungal load without causing severe OPC. Thus, in the oral cavity B lymphocytes contribute to control commensal *C. albicans* carriage by secreting IgA at foci of colonization thereby preventing fungal dysbiosis.

## Introduction

The occurrence of fungal infections is rising and a serious threat to the public health (1), yet this problem is relatively underappreciated by the press, the public and funding agencies. In the United States fungal diseases cost more than $7.2 billion annually, including $4.5 billion from >75,000 hospitalizations and $2.6 billion from ~9 million outpatient visits (2). Very few fungal species cause disease in humans. However, some of these opportunistic fungal pathogens are ubiquitous members of the normal human mycobiome. Indeed, the oral cavity hosts various commensal fungal species (3). As part of the human mycobiome the polymorphic fungus *Candida albicans* colonizes the oral mucosal surface of up to 70% of healthy individuals (4). Due to local or systemic immunosuppression, this fungus is able to proliferate resulting in oral disease, termed oropharyngeal candidiasis (OPC) (5). However, in healthy individuals *C. albicans* causes no harm. In fact, commensal fungi, such as *C. albicans*, are required for microbial community structure, metabolic function, and immune priming (6–8). An imbalance of the mycobiome equilibrium, termed fungal dysbiosis, changes the functional composition, structure, and metabolic activities of the host microbial communities (7). Gain of function dysbiosis (9, 10) may lead to mucosal fungal infection such as OPC. The human host evolved finely tuned innate and adaptive immune responses enabling to control fungal commensal organism (5, 11). Unlike humans mice do not host *C. albicans* in their mycobiome (12). Thus, oral fungal challenge with the commonly used laboratory *C. albicans* strain SC5314 generates an acute immune response in a naive host (13). Since the adaptive immunity plays a critical role in maintaining immune tolerance toward commensal organisms, such as commensal *C. albicans*, understanding its relationship with fungi is critical (14). In an adaptive immunity OPC rechallenge model using a derivate of the pathogenic *C. albicans* strain SC5314, CD4^+^ Th17 cells protect from mucosal *Candida* infection but can be compensated by other IL-17-producing cells in CD4-deficient hosts (15, 16). However, as a commensal, *C. albicans* is in constant interaction with the host epithelium (17). Therefore, lack of incessant fungal exposure limited our advances in understanding antifungal adaptive immune responses at mucosal surfaces in this rechallenge model. Recently, Schonherr *et al*. showed that prolonged oral *C. albicans* colonization depends on the fungal isolate and can be accomplished without immunosuppression of the host thus mimicking the scenario in humans (18). Strikingly, fungal persistence in the oral cavity is independent of a suppressed antifungal immunity since regulatory T cells depletion or deletion of the immune regulatory cytokine IL-10 did not alter the protective type 17 immunity (19). However, tissue-resident memory (T_RM_) Th17 cells prevent uncontrolled outgrowth of the commensal fungus (20).

Immunoglobulin A (IgA) is thought to be a bridge between the innate and adaptive immunity. IgA is predominantly induced in response to colonization with commensal organism therefore maintaining homeostasis via immune exclusion (21–23). Among the production of mucosal antibodies, particularly IgA, by tissue-resident B cells is key to controlling the composition of the microbiome (24). IgA is the dominant antibody isotype in the mucosal immune system, which widely exists in the gastrointestinal tract, respiratory tract, vaginal tract, tears, saliva, and colostrum (25). Immune exclusion is the primary mechanism by which secretory low-affinity IgA (sIgA) blocks microorganisms from attaching to mucosal epithelial cells, thereby preventing colonization, damage, and subsequent invasion (26).

In the present study, we utilized *C. albicans* clinical isolates which are able to persist in the oral cavity without causing disease to analyze adaptive responses to *C. albicans* colonization. We found that oral fungal colonization upregulates adaptive host responses, including the upregulation of the immune network for IgA production. *C. albicans* colonization increased the total salivary and tissue IgA levels, thereby preventing adhesion and invasion of the fungus. Furthermore, B cells, plasmablasts, and plasma cells accumulated to foci of fungal colonization at the epithelial surface. Importantly B cell deficiency and antibody-mediated B lymphocyte depletion increased the commensal *C. albicans* load without causing severe OPC. Thus, in the oral mucosa accumulating B lymphocytes and secreted IgA control commensal *C. albicans* carriage by preventing fungal outgrowth.

## Materials and Methods

### Ethics Statement

All animal work was approved by the Institutional Animal Care and Use Committee (IACUC) of the Lundquist Institute at Harbor-UCLA Medical Center.

### Organisms and Cell Lines

The *C. albicans* strains SC5314 (27), 529L (28), and CA101 (18) were used in the experiments and were grown as described previously (29). The *Streptococcus oralis* strain was purchased from the American Type Culture Collection (ATCC; #35037) and was grown in brain heart infusion broth. The OKF6/TERT-2 oral epithelial cell line (30) was grown as described (31). The OKF6/TERT-2 cells have been authenticated by RNA-Seq (32), and have been tested for mycoplasma contamination. The cell line of murine tongue-derived keratinocytes (TDKs) were kindly provided by S. LeibundGut-Landmann and grown as previously described (33).

### Mouse Model of Oropharyngeal Candidiasis

Six week old male C57BL/6J, B6.129S2-*Ighm*^*tm*1*Cgn*^/J (muMt), and B6.129S7-*Rag1*^*tm*1*Mom*^/J (*Rag1* KO) mice were purchased from Jackson laboratories and housed for 1 week in a pathogen free facility prior infection. The mice were randomly assigned to the infection groups. OPC was induced in mice as described previously (34, 35). Briefly, for inoculation, the animals were sedated, and a swab saturated with 2 × 10^7^ *C. albicans* cells was placed sublingually for 75 min. For colony-forming unit (CFU) enumeration the tongues were harvested, weighed, homogenized and quantitatively cultured. Wild-type (C57BL/6J) mice were sacrificed after 2, 5, 11, and 20 days of infection. muMT and *Rag1* KO mice were sacrificed after 7 days of oral infection with the commensal stains 529L and CA101, respectively. To determine CFUs of commensal outgrowth after systemic immunosuppression wild-type mice were colonized with 529L and CA101. On day 11 and 13 post oral infection mice were given a subcutaneous injection of 25 mg/kg triamcinolone (Kenalog-10, Bristol-Myers Squibb Company). The researchers were not blinded to the experimental groups because the endpoints (oral fungal burden) were an objective measure of disease severity. Saliva was collected at day 11 post infection into chilled tubes after intraperitoneal carbachol injection (100 μl at 10 mg/ml). For antibody depletion, wild-type mice were treated intraperitoneally with 300 μg of anti-mouse B220 (RA3.3A1/6.1, BioXCell) and 300 μg of anti-mouse CD19 (1D3, BioXCell), or isotype controls (2A3, BE0094, BioXCell) on day−1, 4, and 9 relative to infection. For RNA sequencing tongues from 3 Sham-infected and 3 529L colonized mice after 5 and 11 days post infection were processed for analysis.

### RNA Sequencing

Total RNA was isolated as described elsewhere (32) and RNA sequencing was performed by Novogene Corporation Inc. (Sacramento, USA). mRNA was purified from total RNA using poly-T oligo-attached magnetic beads. To generate the cDNA library the first cDNA strand was synthesized using random hexamer primer and M-MuLV Reverse Transcriptase (RNase H^−^). Second strand cDNA synthesis was subsequently performed using DNA Polymerase I and RNase H. Double-stranded cDNA was purified using AMPure XP beads and remaining overhangs of the purified double-stranded cDNA were converted into blunt ends via exonuclease/polymerase. After 3' end adenylation a NEBNext Adaptor with hairpin loop structure was ligated to prepare for hybridization. In order to select cDNA fragments of 150~200 bp in length, the library fragments were purified with the AMPure XP system (Beckman Coulter, Beverly, USA). Finally, PCR amplification was performed and PCR products were purified using AMPure XP beads. The samples were read on an Illumina NovaSeq 6000 with ≥20 million read pair per sample.

### Downstream Data Processing

Downstream analysis was performed using a combination of programs including STAR, HTseq, and Cufflink. Alignments were parsed using Tophat and differential expressions were determined through DESeq2. KEGG enrichment was implemented by the ClusterProfiler. Gene fusion and difference of alternative splicing event were detected by Star-fusion and rMATS. The reference genome of *Mus musculus* (GRCm38/mm10) and gene model annotation files were downloaded from NCBI/UCSC/Ensembl. Indexes of the reference genome was built using STAR and paired-end clean reads were aligned to the reference genome using STAR (v2.5). HTSeq v0.6.1 was used to count the read numbers mapped of each gene. The FPKM of each gene was calculated based on the length of the gene and reads count mapped to it. FPKM, Reads Per Kilobase of exon model per Million mapped reads, considers the effect of sequencing depth and gene length for the reads count at the same time (36). Differential expression analysis was performed using the DESeq2 R package (2_1.6.3). The resulting *P*-values were adjusted using the Benjamini and Hochberg's approach for controlling the False Discovery Rate (FDR). Genes with an adjusted *P*-value < 0.05 found by DESeq2 were assigned as differentially expressed (cutoff fold change 1.5, Supplementary Table 1). To allow for log adjustment, genes with 0 FPKM are assigned a value of 0.001. Correlation were determined using the cor.test function in R with options set alternative = “greater” and method = “Spearman.” To identify the correlation between the differences, we clustered different samples using expression level FPKM to see the correlation using hierarchical clustering distance method with the function of heatmap, SOM (Self-organization mapping) and kmeans using silhouette coefficient to adapt the optimal classification with default parameter in R. We used clusterProfiler R package to test the statistical enrichment of differential expression genes in KEGG pathways. The high-throughput sequencing data from this study have been submitted to the NCBI Sequence Read Archive (SRA) under accession number PRJNA657562.

### Adhesion and Invasion Assay

Adhesion of *C. albicans* and invasion into oral epithelial cells was quantified by a differential fluorescence assay as described previously (31, 37). Briefly, OKF6/TERT-2 cells were grown to confluency on fibronectin-coated circular glass coverslips in 24-well tissue culture plates. *C. albicans* was incubated with 100 μg/ml sIgA (BioRad) for 30 min. The epithelial cells were infected with 2 × 10^5^ yeast-phase *C. albicans* SC5314 cells per well (multiplicity of infection; MOI 1) and incubated for 2.5 h, after which they were fixed, stained, and mounted inverted on microscope slides. The coverslips were viewed with an epifluorescence microscope, and the number of endocytosed organisms per high-power field was determined, counting at least 100 organisms per coverslip. Each experiment was performed at least three times in triplicate. To determine the effect of saliva from infected mice on *Candida* adhesion and invasion saliva from 3 mice was pooled, diluted 1:1 in PBS and incubated with *C. albicans* before added to 2 × 10^5^ the murine keratinocyte cells for 2.5 h. Fungal adhesion and invasion were determined as described above.

### Cytokine and Chemokine Measurements *in vitro*

Cytokine levels in culture supernatants were determined as previously described (38). Briefly 2 × 10^5^ OKF6/TERT-2 cells in a 24-well plate were infected with *C. albicans* SC5314 at a MOI of 5. Prior to infection *C. albicans* was coated with sIgA as described above. After 8 h of infection, the supernatant was collected, clarified by centrifugation and stored in aliquots at −80°C. The concentration of inflammatory cytokines and chemokines in the medium was determined using the Luminex multipex assay (R&D Systems). Each condition was tested in three independent experiments.

### IgA ELISA

To determine IgA levels saliva was collected as described above, and diluted 1:10 in PBS. Tongue homogenates were collected as described previously (38) and analyzed for IgA levels using manufactures instructions (IgA Elisa Mouse, Invitrogen).

### Immunofluorescence

To determine B220^+^ and IgA^+^ cell localization *in vivo*, 30–50-μm-thick sections of OCT-embedded tongues were fixed with cold acetone. Next, the cryosections were rehydrated in PBS and then blocked using BSA. To detect IgA (FITC, mA-6E1, Invitrogen) or B220 (Alexa 488, RA3-6B2, Biolegend) positive cells sections were incubated with 1:50 diluted antibody overnight. To detect *C. albicans*, the sections were also stained with an anti-*Candida* antiserum (Biodesign International) conjugated with Alexa Fluor 568 (Thermo Fisher Scientific) for 1 h. To visualize the nuclei, the cells were stained with DAPI (4′,6-diamidino-2-phenylindole). The sections (z-stack) were imaged by confocal microscopy. To enable comparison of fluorescence intensities among slides, the same image acquisition settings were used for each experiment.

### Salivary IgA Binding to *S. oralis*

10^8^ *S. oralis* were incubated with 10 μl saliva for 45 min isolated from Sham-infected mice, or mice infected with *C. albicans* 11 days post infection. Bacteria were washed 3 times before stained with anti-IgA antibody (FITC, mA-6E1). The stained organisms were analyzed on FACSymphony system (BD Biosciences), and the data were analyzed using FACS Diva (BD Biosciences) and FlowJo software (Treestar).

### Flow Cytometry of B Lymphocytes

To determine the number of B lymphocytes in the mouse tongues single cell suspension were generated as described previously (39, 40). Briefly, mice were orally infected with *C. albicans* as described above. After 11 days of infection, the animals were administered a sublethal anesthetic mix intraperitoneally. The thorax was opened, and a part of the rib cage removed to gain access to the heart. The vena cava was transected and the blood was flushed from the vasculature by slowly injecting 10 ml PBS into the right ventricle. The tongue was harvested and cut into small pieces in 100 μl of ice-cold PBS. 1 ml digestion mix (4.8 mg/ml Collagenase IV; Worthington Biochem, and 200 μg/ml DNase I; Roche Diagnostics, in 1x PBS) was added after which the tissue was incubated at 37°C for 45 min. The resulting tissue suspension was then passed through a 100 μm cell strainer. The single-cell suspensions were incubated with rat anti-mouse CD16/32 (2.4G2; BD Biosciences) for 10 min in FACS buffer at 4°C to block Fc receptors. For staining of surface antigens, cells were incubated with fluorochrome-conjugated (FITC, PE, PE-Cy7, allophycocyanin [APC], APC-eFluor 780) antibodies against mouse TER-119 (TER-110, BioLengend), CD326 (G8.8, BioLegend), CD11b (M1/70, BioLegend), Gr-1 (RB6-8C5, BioLegend), CD3 (17A2, BioLegend), CD8a (53-6.7, BioLegend), CD4 (GK1.5, BioLegend), CD45R/B220 (9RA3-6B2, BioLegend), CD19 (6D5, BioLegend), CD138 (281-2, BioLegend). After washing with FACS buffer, the cell suspension was stained with a LIVE/DEAD fluorescent dye (7-AAD; BD Biosciences) for 10 min. For intracellular staining, cell viability was determined using BD Horizon Fixable Viability Stain 780 (BD Biosciences) followed by Cytofix/Cytoperm (BD Biosciences) treatment staining with KI67 antibody (16A8; BioLegend). The stained cells were analyzed on FACSymphony system (BD Biosciences), and the data were analyzed using FACS Diva and FlowJo software. Only single cells were analyzed, and cell numbers were quantified using PE-conjugated fluorescent counting beads (Spherotech).

## Results

### Commensal *C. albicans* Isolates Persist in the Oral Cavity and Cause OPC During Systemic Immunosuppression

**Figure 1:**
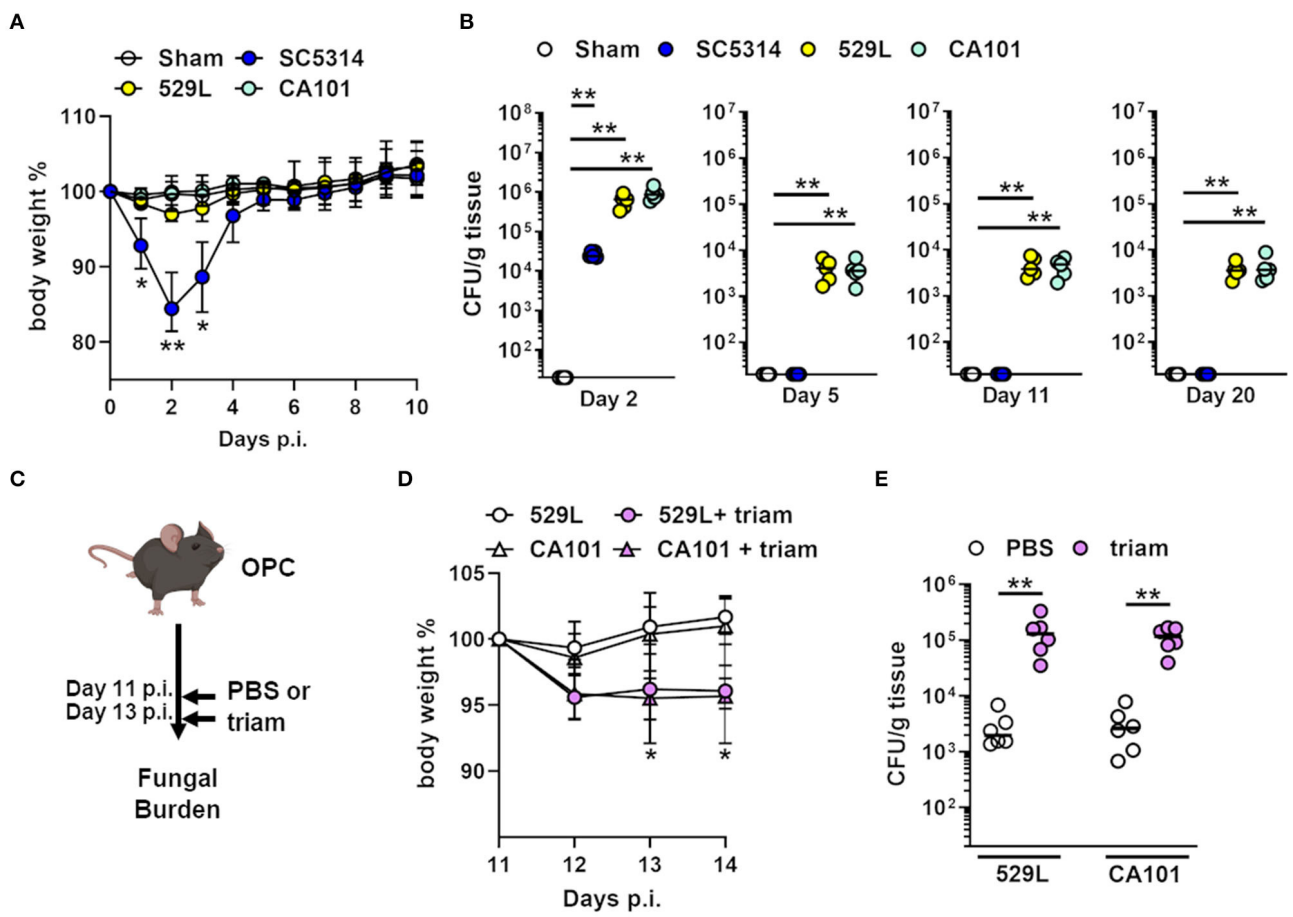
Commensal *C. albicans* strains cause OPC during immunosuppression. **(A)** Body weight (mean± range) of mice orally infected with indicated strains. ^*^P < 0.05, ^**^P < 0.01 (*n* = 10; Mann–Whitney). **(B)** Oral fungal burden of wild-type mice infected with indicated strains. Results are median of two independent experiments (*n* = 5). ^**^P < 0.01 (Kruskal-Wallis). The y-axis is set at the limit of detection (20 CFU/g tissue). **(C)** Mouse model of immunosuppressed OPC during *C. albicans* colonization. Triam, triamcinolone. **(D)** Body weight (mean ± range) of mice starting day 11 during fungal colonization and immunosuppression. Day 11 post infection set as 100%. **(E)** Oral fungal burden of wild-type mice infected with indicated commensal strains 11 days post oral infection. Results are median of two independent experiments (*n* = 6). ^**^P < 0.01 (Mann-Whitney). The y-axis is set at the limit of detection (20 CFU/g tissue).

To investigate if oral commensal *C. albicans* isolates (18, 19) induce adaptive immune responses we infected wild-type mice orally with a pathogenic strain SC5314 and two commensal *C. albicans* isolates 529L and CA101, respectively. While oral infection with the pathogenic *C. albicans* strain led to significant body weight loss and rapid clearance from the oral cavity, the commensal strains 529L and CA101 colonized the oral mucosa and persisted for over 20 days without inducing significant weight loss in the host (Figures 1A,B). Although the commensal strains persist in the oral cavity, we noticed a significant decline between day 2 and 5 post infection (Figure 1B) similar what has been reported previously (20). Despite the fact that distinct *C. albicans* clinical isolates are able to persist in the oral cavity it is unclear if these strains can outgrow and induce severe oral disease. The induction of prolonged OPC in naive mice has been extensively studied using corticosteroids (31, 34, 35, 37, 38). To test the potential of commensal fungal outgrowth during immunosuppression we colonized mice with the *C. albicans* strains 529L and CA101 for 11 days and induced systemic immunosuppression using triamcinolone (Figure 1C). The administration of triamcinolone resulted in significant body weight loss and >40-fold increase in oral fungal burden (Figures 1D,E). These data indicate that systemic immunosuppression leads to fungal outgrowth of colonizing commensal *C. albicans* in the oral cavity.

### *C. albicans* Oral Colonization Induces Upregulation of Adaptive Immune Response Signatures

**Figure 2:**
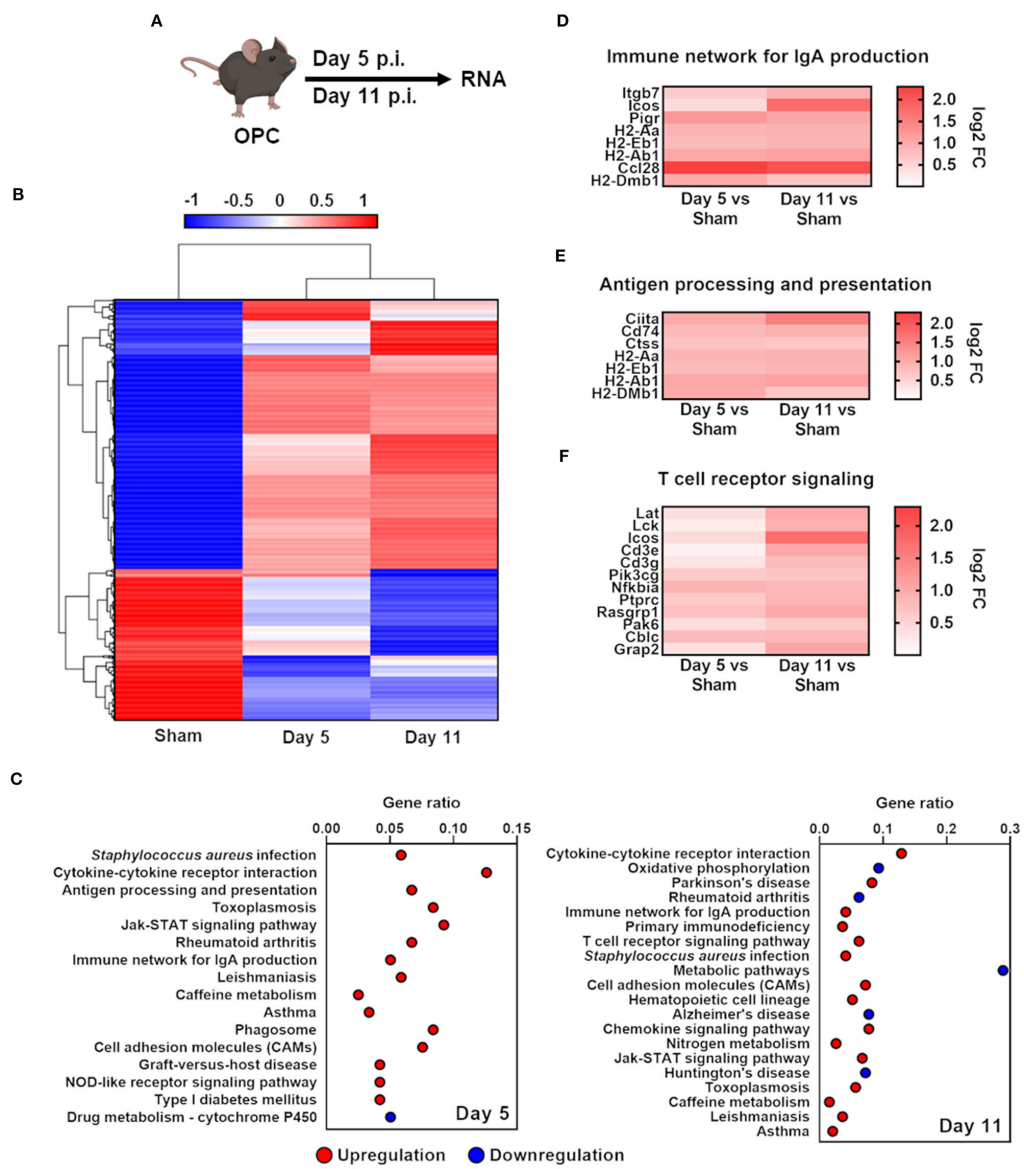
*C. albicans* oral colonization induces upregulation of adaptive response pathways in immunocompetent mice. **(A)** Scheme of infection with 529L or Sham and time points of RNA isolation. **(B)** Heat map showing hierarchical clustering of *C. albicans* colonization with 529L in the oral cavity after 5 and 11 days of genes with a fold change of FC >1.5. Red denotes genes with high expression levels, and blue denotes genes with low expression levels. The color ranging from red to blue indicates log10 (FPKM+1) value from highest to lowest. **(C)** Identified pathways of enriched genes FC > 1.5, adjusted *P* < 0.05. Shown is the gene ratio (Genes of pathway/all different expressed genes; padj ≤ 0.5). **(D–F)** Heat map of enriched genes of corresponding pathways.

To obtain genome-wide information about the host response to commensal *C. albicans* colonization in the oral cavity, we performed RNA sequencing. Given the fact that the tested persisting *C. albicans* strains 529L and CA101 behaved similarly in the mouse model of OPC (Figure 1) we used 529L (41) as a representative commensal strain to assess the transcriptional commensal-specific host response landscape at 5 and 11 days post-infection compared to Sham-infected mice (Figure 2A). Using unsupervised hierarchical clustering, we found oral *C. albicans* colonization leads to robust changes and dynamic host responses (Figure 2B). *C. albicans* colonized mice were clustered in one group, with four broad gene clusters. Pathway analysis revealed an upregulation of adaptive host responses due to *C. albicans* persistence (Figure 2C), including the upregulation of the immune network for IgA production, antigen processing and presentation, and T cell receptor signaling (Figures 2D–F). Thus, oral fungal colonization leads to a robust induction of adaptive immune responses in *C. albicans* immunological naive mice.

### Oral *C. albicans* Colonization Upregulates Salivary IgA and Induces Migration of IgA Secreting Cells in the Oral Epithelial Layer

**Figure 3:**
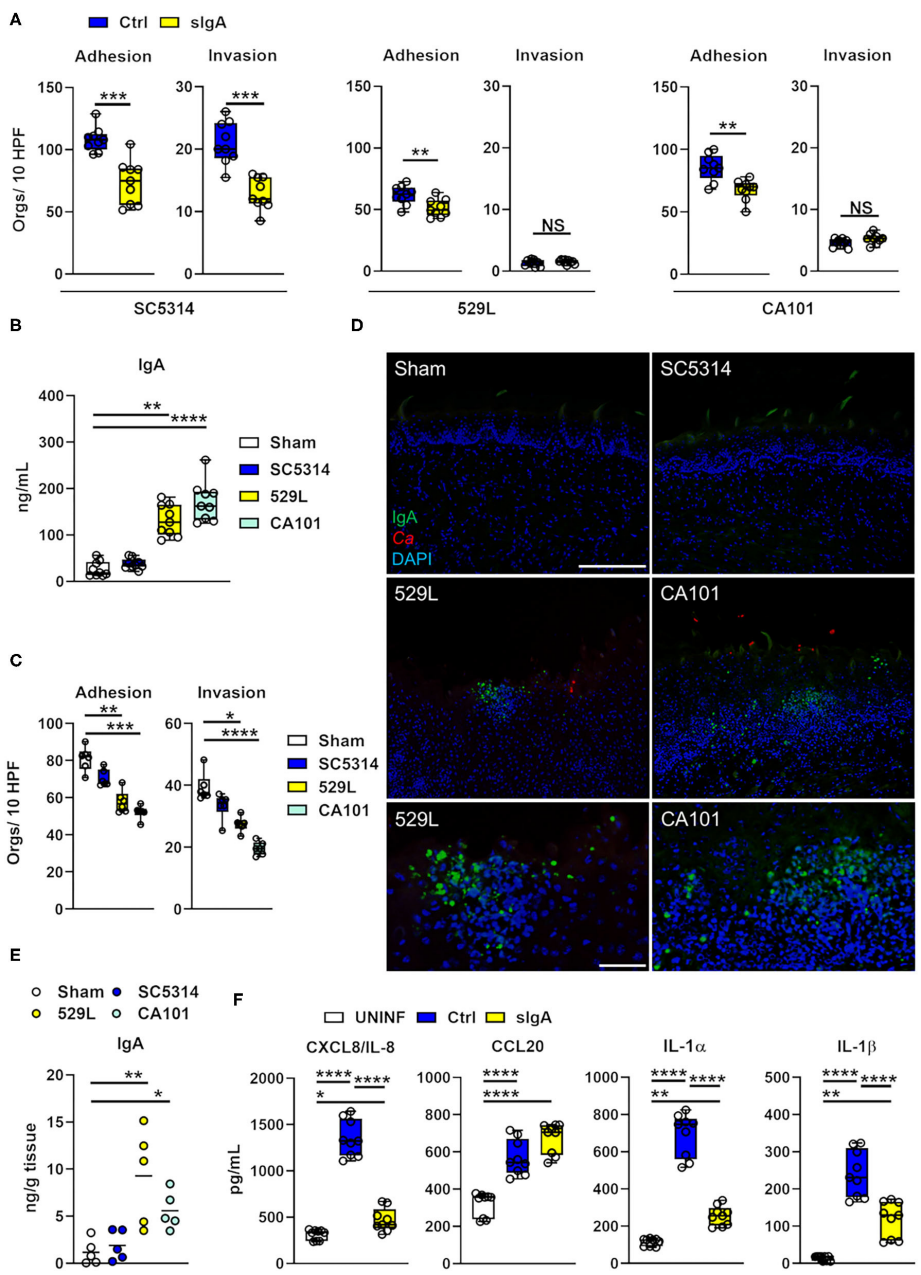
Oral fungal colonization upregulates mucosal IgA preventing *C. albicans* epithelial adhesion and invasion. **(A)** Indicated *C. albicans* strains were incubated with sIgA prior to infection of the OKF6/TERT-2 oral epithelial cell line. The cells were infected for 2.5 h, after which the number of adhered and invaded organisms was determined using a differential fluorescence assay. Box Whisker plot shows three experiments, each performed in triplicate. Orgs/10 HPF, organisms per 10 high-power fields; Ctrl, control. Statistical significance was determined using Mann-Whitney test (^*^P < 0.05; ^**^P < 0.01). **(B)** Total IgA amounts in saliva (diluted 1:10) determined by ELISA. Saliva was collected after 11 days of infection with indicated *C. albicans* strains. Three independent experiments performed in triplicate. ^**^P < 0.01; ^****^P < 0.0001 (Kruskal-Wallis). **(C)** Indicated *C. albicans* strain was incubated with saliva of infected with prior infection of the murine keratinocyte cell line. The cells were infected for 2.5 h, after which the number of adhered and invaded organisms was determined using a differential fluorescence assay. Box Whisker plot shows three experiments, each performed in dublicate. Orgs/10 HPF, organisms per 10 high-power fields. Statistical significance was determined using Kruskal-Wallis test (^*^P < 0.05; ^**^P < 0.01; ^***^P < 0.001; ^****^P < 0.0001). **(D)** Immunofluorescence of IgA in tongues 11 days post oral infection with indicated strains. IgA is shown in green, and *C. albicans* (*Ca*) in red. DAPI (blue) visualizes the tissue. Upper, middle panel scale bar 200 μm. Lower panel scale bar 50 μm. **(E)** Total IgA amounts in tongue homogenates determined by ELISA. Tongues were collected after 11 days of infection with indicated *C. albicans* strains; *N* = 5. ^*^P < 0.05; ^**^P < 0.01 (Kruskal-Wallis). **(F)** Indicated *C. albicans* strains were incubated with sIgA prior to infection of the OKF6/TERT-2 oral epithelial cell line. The cells were infected for 8 h after which CXCL8/IL-8, CCL20, and IL-1α/β were determined in the supernatant. Box Whisker plot shows three experiments triplicate. Uninf; uninfected; Ctrl, control. Statistical significance was determined using Kruskal-Wallis (^*^P < 0.05; ^**^P < 0.01; ^****^P < 0.0001).

sIgA inhibits the adhesion of *C. albicans* hyphae to polystyrene (42). Since epithelial adhesion and invasion are required for *C. albicans* oral infection (4, 43) we determined if the physiological sIgA concentration found in healthy individuals (44) prevents adhesion of *C. albicans* to and invasion of human oral epithelial cells. Incubation of the pathogenic *C. albicans* strain SC5314 with sIgA decreased adherence and invasion of OKF6-TERT2 oral epithelial cells (Figure 3A) and adhesion of the commensal strains 529L and CA101, respectively. Of note, the persisting *C. albicans* strains showed remarkable reduction of epithelial adhesion and invasion compared to the pathogenic strain SC5314 (Figure 3A). High slgA levels are found in various secretory fluids, including saliva (45). Therefore, we measured IgA levels in the saliva of commensal and pathogenic strain infected mice, and found that *C. albicans* oral commensal colonization with 529L and CA101 increased the abundance of total IgA, while infection with the pathogenic strain SC5314 did not upregulate salivary IgA compared to Sham-infected mice (Figure 3B). Next, we measured if the saliva from Sham- or *C. albicans*-infected mice is able to prevent fungal adhesion to and invasion of murine tongue-derived keratinocytes (33) using the pathogenic strain SC5314. Saliva from commensal colonized mice was able to prevent epithelial adhesion and invasion compared to saliva from Sham-infected mice or mice infected with the pathogenic strain SC5314 (Figure 3C). The majority of the hosts entire pool of activated B cells is located near the mucosa as well as exocrine glands (46). Thus, we assessed tissue distribution of IgA^+^ cells in Sham-infected mice, or mice infected either the pathogenic strain SC5314 or the commensal strains 529L and CA101, respectively. IgA^+^ cells accumulated exclusively in oral epithelial and submucosal layers of mice colonized with the commensal *C. albicans* strains 529L and CA101 (Figure 3D). Consistent with this observation total IgA levels increased in tissue homogenates of commensal *C. albicans* colonized mice after 11 days of infection (Figure 3E). By secreting proinflammatory cytokines and chemokines, oral epithelial cells are vital for limiting fungal proliferation during OPC (4, 5). *In vivo* commensal *C. albicans* strains fail to induce a strong acute inflammatory response (18). Therefore, we assessed oral epithelial proinflammatory cytokine/chemokine production in the presence and absence of sIgA. We found that binding of sIgA to *C. albicans* dampend the secretion of the inflammatory mediators CXCL8/IL-8, IL-1α, and IL-1β while CCL20 secretion was unaffected (Figure 3F) suggesting that *C. albicans*-IgA interactions reduces a subset of the proinflammatory epithelial response. Collectively, our data suggest that fungal colonization upregulates salivary and tissue IgA production by inducing migration of IgA^+^ cells in close proximity of colonizing fungi and thereby preventing adhesion and invasion of fungi in the oral cavity.

### *C. albicans* Oral Colonization Increases Cross-Specific IgA Levels in the Oral Cavity

**Figure 4:**
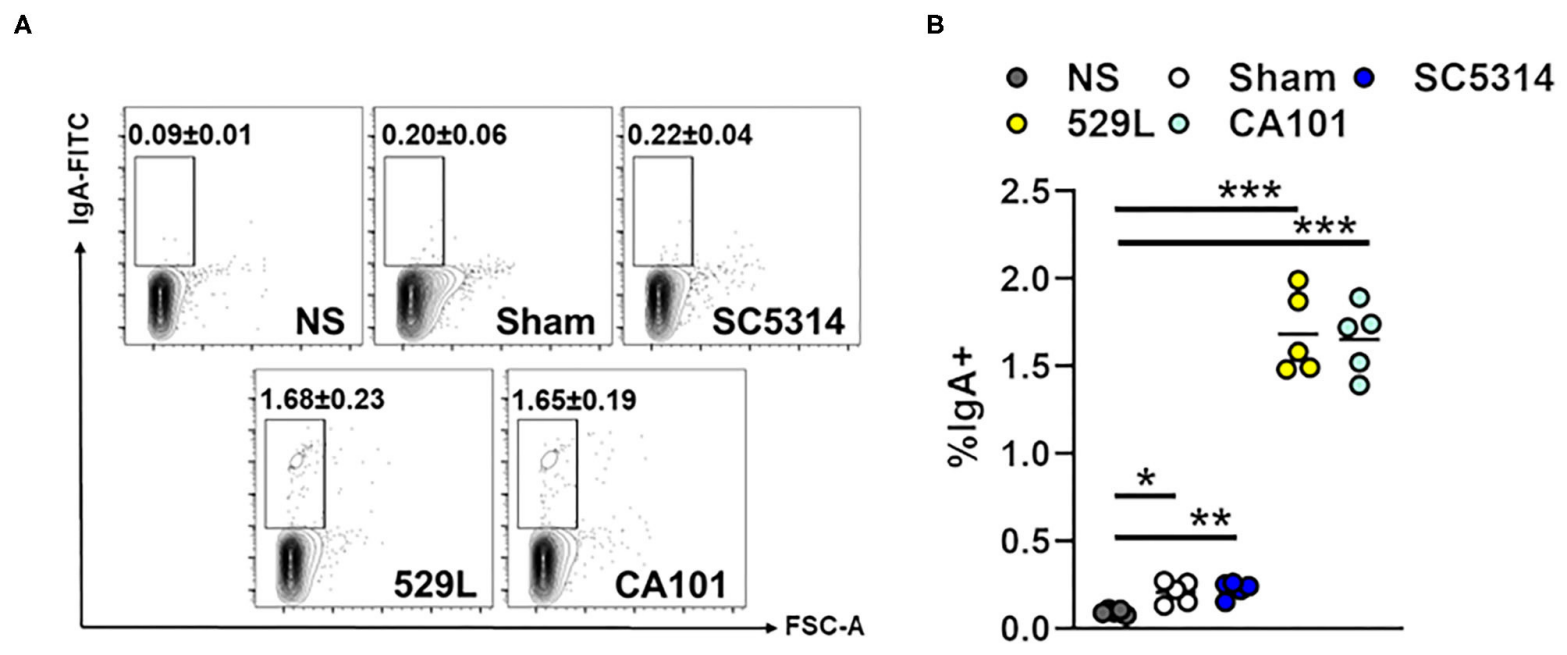
Fungal colonization increases cross-specific IgA levels. **(A)** Representative flow plots of salivary IgA bound to *S. oralis*. NS, No Saliva. **(B)** The percentage of IgA^+^ S. *oralis*. Results are median of two independent experiments with 5 mice per group. ^*^P < 0.05, ^**^P < 0.01, ^***^P < 0.001 (Kruskal-Wallis).

Secretory IgA can have a measurable reactivity to a diverse subset of the microbiota. IgAs interact with commensal organisms by canonical Fab-dependent and non-canonical carbohydrate-dependent binding (47). Therefore, we tested if salivary IgA of infected mice binds to the common oral commensal *Streptococcus oralis* (48). While IgA from Sham-infected and mice infected with the pathogenic strain SC5314 were able to bind *S. oralis* (Figures 4A,B) IgA binding to *S. oralis* increased >7-fold when the bacteria were incubated with saliva from commensal colonized mice. Thus, oral colonization with commensal *C. albicans* increases total levels of cross-specific IgAs.

### Mucosal B Lymphocytes Expand During Oral Fungal Colonization

**Figure 5:**
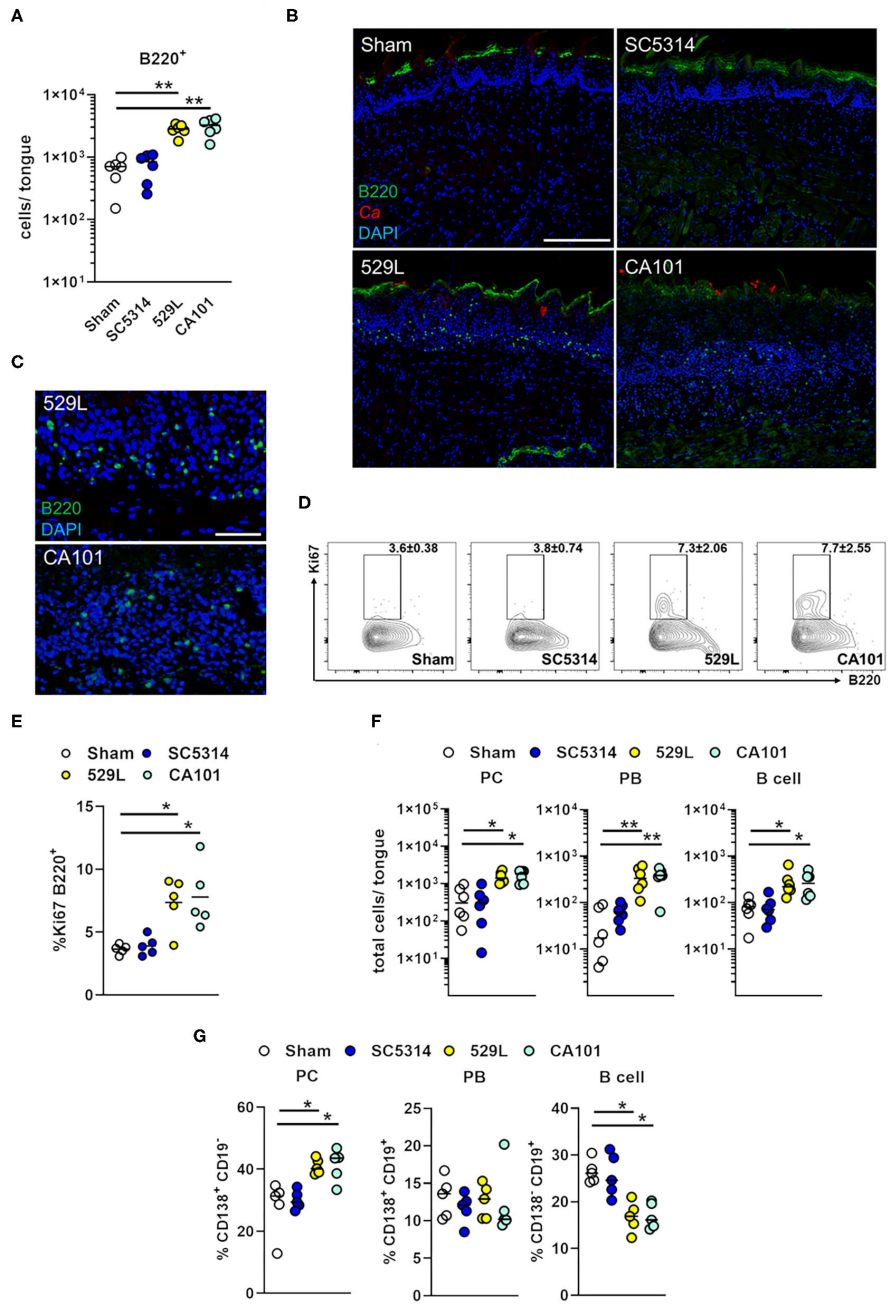
B lymphocytes accumulate in the oral epithelial and submucosal layers during fungal colonization. **(A)** B220^+^ cell infiltration in tongues of immunocompetent wild-type mice after 11 days of infection with indicated *C. albicans* strains (*n* = 6). B220^+^ cells were gated on singlets live CD4^−^ CD8^−^ CD11b^−^ Gr-1^−^ TER-119^−^ EpCam^−^. Results are median from combined results of two independent experiments. ^**^P < 0.01 (Kruskal-Wallis). **(B)** Immunofluorescence of B220^+^ cells in tongues 11 days post oral infection with indicated strains. B220 is shown in green, and *C. albicans* (*Ca*) in red. DAPI (blue) visualizes the tissue. Scale bar 200 μm. **(C)** Scale bar 50 μm. **(D)** Representative flow plots of Ki67^+^ B220^+^ cells in the tongue after 11 days of infection. Cells were gated on singlets live B220^+.^ **(E)** The percentage of Ki67^+^ B220^+^ cells. Results are median of a single experiment with 5 mice per group. ^*^P < 0.05 (Kruskal-Wallis). **(F)** Total numbers of plasma cells (PC; CD19^−^ CD138^+^), plasmablasts (PB; CD19^+^ CD138^+^), and mature B cells (CD19^+^ CD138^−^) were determined after 11 days post infection with indicated strains. Cells were gated on singlets live B220^+^ CD4^−^ CD8^−^ CD11b^−^ Gr-1^−^ TER-119^−^ EpCam^−^. Results are median from combined results of two independent experiments (*n* = 6). ^*^P < 0.5; ^**^P < 0.01 (Kruskal-Wallis). **(G)** Percentage of plasma cells, plasmablasts, and mature B cells were determined after 11 days post infection with indicated strains. ^*^P < 0.5 (Kruskal-Wallis).

Following the initiation of an immune response, B lymphocytes preferentially migrate back to initial sites of antigen encounter (49). We therefore determined B lymphocyte distribution in the oral cavity of infected mice. B lymphocytes (B220^+^ cells) were enriched during oral commensal colonization with 529L and CA101 in the whole tongue (Figure 5A, Supplementary Figure 1A) localized in the oral epithelial and submucosal layers during fungal colonization (Figures 5B,C). The expansion of B220^+^ cells could be due to local proliferation (50). Therefore, WT mice were infected orally and intracellular Ki67 was measured by flow cytometry. On day 11, Ki67^+^B220^+^ cells were more frequent in the infected oral mucosa of mice infected with the commensal *C. albicans* strains compared to Sham controls or pathogenic infected mice (Figures 5D,E). Thus, the expansion of B220^+^ cells during *Candida* oral colonization can be accounted for by proliferation at the site of fungal persistence. Next we determined B lymphocyte subpopulations including plasma cells (CD19^−^ CD138^+^), plasmablasts (CD19^+^ CD138^+^), and mature B cells (CD19^+^ CD138^−^) in Sham-infected mice, mice infected with the pathogenic strain SC5314, or the commensal strains 529L and CA101 (Supplementary Figure 1B). We found that oral colonization with the commensal strains 529L and CA101 increased the tissue distribution of plasma cells, plasmablasts, and mature B cells, while no difference in B lymphocyte populations could be observed when mice were infected with the pathogenic *C. albicans* strain SC5314 compared to Sham-infected mice (Figure 5F). Although the total B lymphocyte numbers increased during commensal colonization (Figure 5F) the plasma cell population expanded and the CD138^−^ CD19^+^ B cell frequency decreased in commensal colonized mice compared to Sham-infected mice (Figure 5G). Thus, oral fungal colonization results in expansion of B lymphocytes.

### Mucosal B Lymphocytes Control Commensal Fungal Load in the Oral Cavity

**Figure 6:**
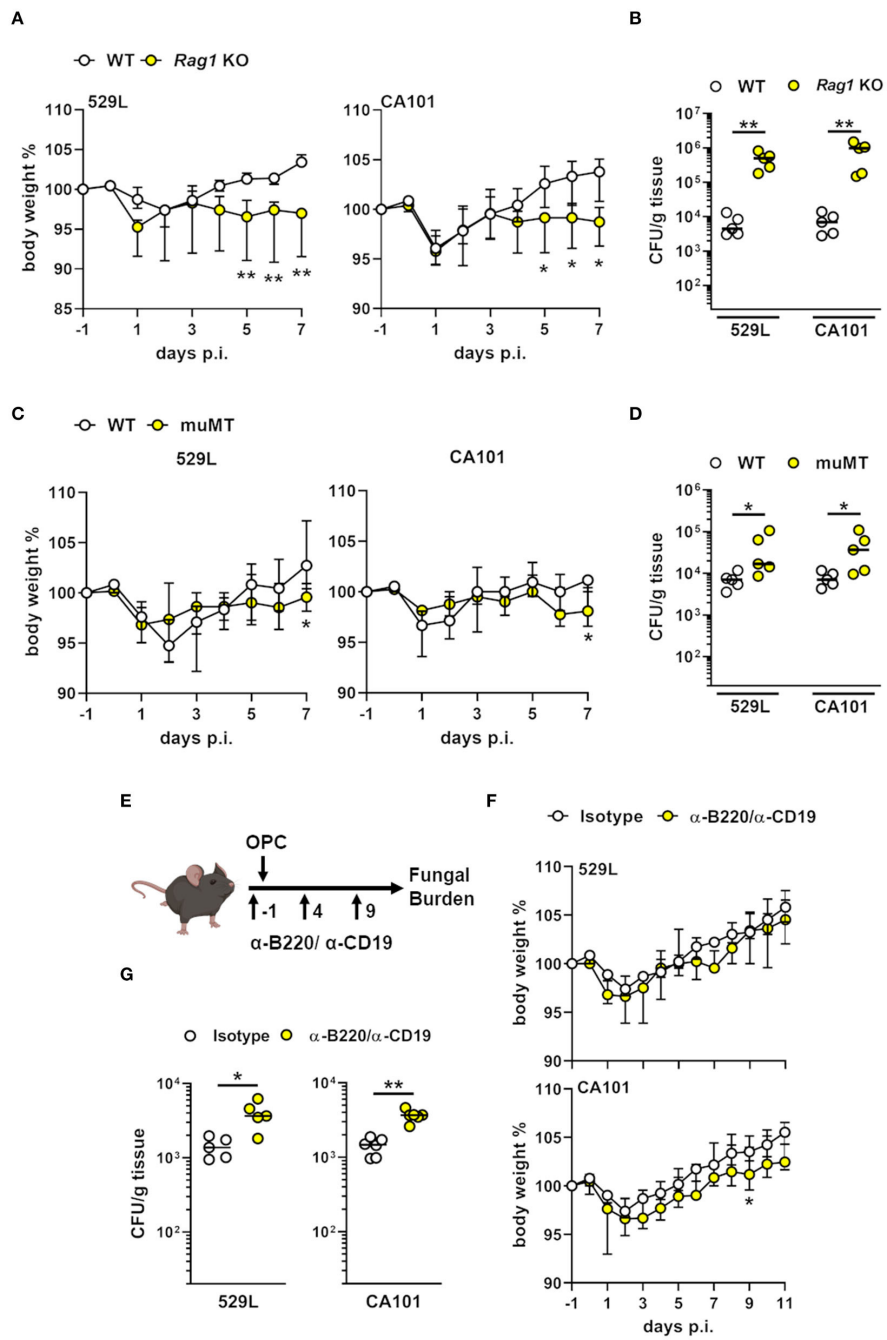
B lymphocytes contribute to commensal control in the oral cavity. **(A)** Body weight of Rag1 deficient and wild-type mice during oral commensal *C. albicans* colonization. ^*^P < 0.05; ^**^P < 0.01 (*n* = 5; Mann-Whitney). **(B)** Oral fungal burden of *Rag1* KO and WT mice infected with indicated strains. Results are median of a single experiment (*n* = 5). ^**^P < 0.01 (Mann-Whitney). The y-axis is set at the limit of detection (20 CFU/g tissue). **(C)** Body weight of muMT and wild-type mice during oral commensal *C. albicans* colonization. ^*^P < 0.05 (*n* = 5; Mann-Whitney). **(D)** Oral fungal burden of muMT and WT mice infected with indicated strains. Results are median of a single experiment (*n* = 5). ^*^P < 0.05 (Mann-Whitney). The y-axis is set at the limit of detection (20 CFU/g tissue). **(E)** Scheme B lymphocyte depletion during commensal OPC using anti-B220/CD19 antibodies. **(F)** Body weight of B lymphocyte depleted and isotype control mice. ^**^P < 0.01 (*n* = 5–6; Mann-Whitney). **(G)** Oral fungal burden 11 days post infection of B lymphocyte depleted and isotype control mice infected with commensal *C. albicans* strains 529L and CA101. Results are median of 5–6 mice per group from two independent experiments. ^*^P < 0.05; ^**^P < 0.01 (Mann-Whitney).

Mice with Rag1 deficiency, the absence of endogenous B- and T cells, show increased susceptibility to oral fungal infection by the pathogenic *C. albicans* strain SC5314 (15). To determine the effect of Rag1 deficiency during commensal colonization we infected WT and *Rag1* KO mice with the commensal strains 529L and CA101. After 5 days of infection Rag1 KO mice lost significantly more body weight compared to WT mice (Figure 6A). Next, we determined the oral fungal burden after 7 days of infection. *Rag1* KO mice had an increase in fungal burden by >100-fold (Figure 6B) (20). Since *Rag1* KO mice lack B- and T cells, we determined the importance of B lymphocytes during oral fungal colonization (51). In a mouse model of fungal asthma, mice lacking the Ig mu-chain (muMT) produce IgG and IgE, but not IgA (52). Therefore, we infected WT and muMT mice with the commensal *C. albicans* strains 529L and CA101, respectively. muMT lost slightly more body weight after 7 days post infection compared to WT mice (Figure 6C). Since the IgA pathway was already induced after 5 days of commensal colonization (Figure 2C) we determined the oral fungal after 7 days post oral inoculation. muMT mice either colonized with 529L or CA101 had an increased fungal burden by >2 to 5-fold (Figure 6D). In a different approach we treated mice with B220/CD-19 antibodies during commensal oral colonization (Figure 6E) thereby reducing mucosal B220^+^ cells (Supplementary Figure 2). B220/CD-19 depletion had minimal effect on body weight of colonized mice (Figure 6F), but increased the oral fungal load by >3-fold after 11 days of colonization (Figure 6G). Thus, the absence of B lymphocytes or the lack of IgA results in commensal *C. albicans* dysbiosis in the oral cavity.

## Discussion

Healthy individuals have a protective *Candida*-specific mucosal immunity which limits fungal proliferation, invasion, and therefore preventing disease (5). Besides the innate immune response, the adaptive immunity to *C. albicans* is crucial to control mucosal fungal outgrowth (11). T cells are an integral component of the antifungal adaptive immune response and provide direct and indirect means of controlling fungal proliferation. Individuals with mutations in the Th17 pathway suffer from chronic mucocutaneous candidiasis (CMC) (53–55). Similarly to humans, mice exposed to *C. albicans* generate long-term adaptive Th17 cell responses that confer additional protection from infection (15, 56). In mice oral fungal persistence is independent of a suppressed antifungal immunity but requires tissue-resident memory Th17 cells to maintain stable fungal colonization (19, 20).

**Figure 7:**
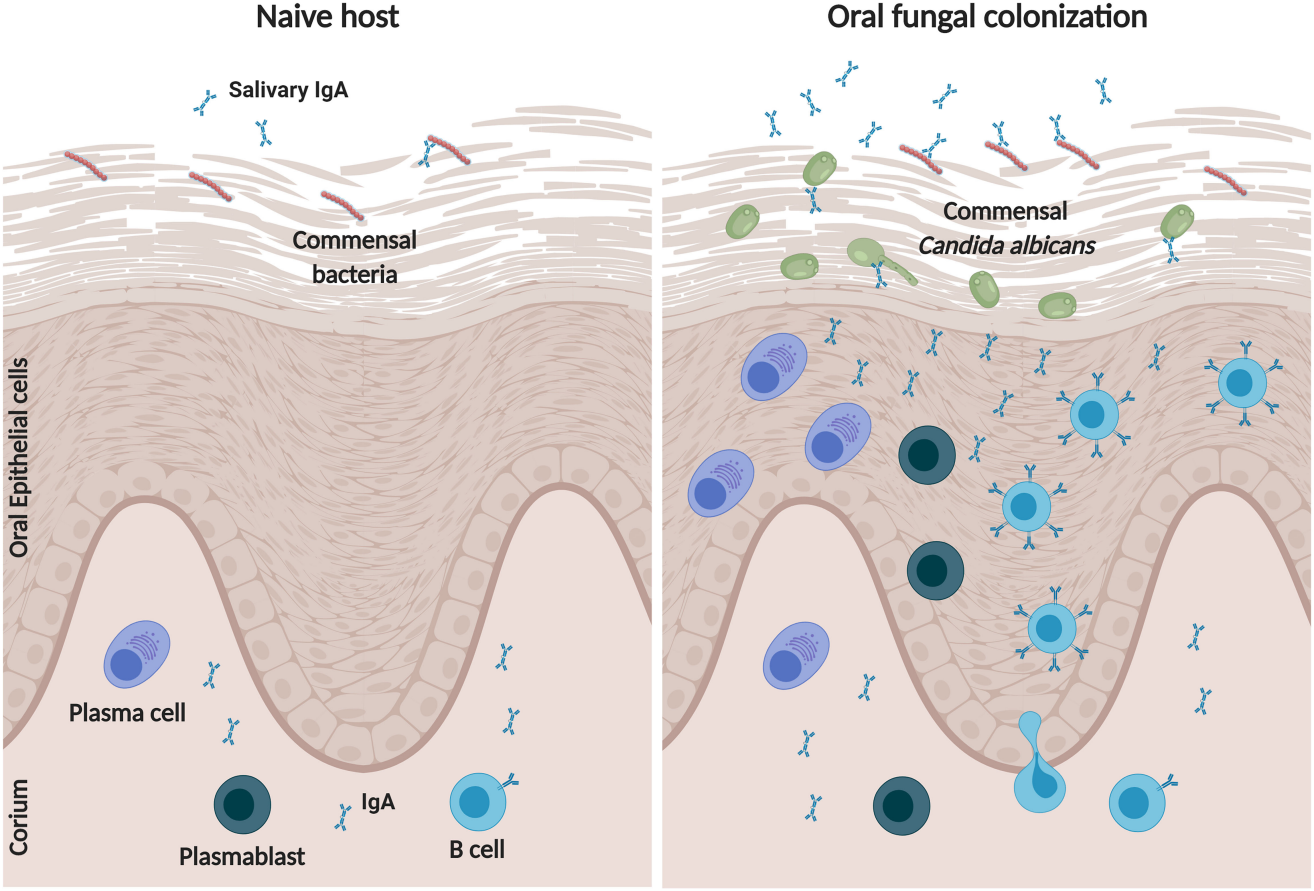
Model of B lymphocyte responses during commensal *C. albicans* colonization in the oral cavity. In a naive host, salivary and tissue IgA binds to commensal bacteria, which in turn regulates mucosal immunity and microbial compostion. During colonization with commensal *C. albicans*, IgA^+^ B cells, in particular mature B cells, plasmablasts and plasma cells, migrate into the oral epithelium and submucosal layers, where they increase the production of polyspecifc-IgAs. The IgA bound to *C. albicans* will reduce fungal adherence and invasion resulting in a dampened proinflammatory response. Created with BioRender.com.

By utilizing persisting commensal *C. albicans* clinical isolates we show that oral colonization generates mucosal adaptive immune response signatures, including antigen processing and presentation, and T cell receptor signaling. In the oral mucosa monocyte-dependent and tissue-resident dendritic cells (DCs) orchestrate the antigen-specific T cell priming toward pathogenic *C. albicans* (57). Secretory antibodies of the IgA class released by effector B cells, including plasma cells, form the first line of immune protection against pathogens and antigens at mucosal surfaces linking the innate and adaptive host immunity (22, 58). Furthermore, mucosal IgA governs quantitative and qualitative control of commensal composition (59). Our data indicates that oral persistence of commensal *C. albicans* stimulates accumulation of B lymphocytes, including plasmablasts and plasma cells, to sites of fungal colonization, where these cells upregulate IgA production (Figure 7). This finding is in agreement with earlier reports showing that that oral mucosal defense against *Candida* involves innate phagocytes, T and B cell recruitment, as well as local antibody production with a prominent IgA component (60, 61). In a mouse model, oral fungal persistence is associated with a weakened proinflammatory host response compared to the pathogenic *C. albicans* strain SC5314 thus preventing *C. albicans* elimination at the onset of colonization (18, 19). While pattern recognition of fungi induces a strong epithelial proinflammatory response (38, 43, 62) the IgA-*Candida* interaction dampens this robust innate response by inhibiting epithelial adherence and invasion of the fungus. Here we show that immune exclusion is a result of *Candida*-IgA interactions *in vitro*. However, the *in vivo* mechanism remains unclear. A recent study showed that IgA-mediated pathogen cross-linking enchains the organism, thereby preventing separation after division resulting in clumping (63). This enchained growth accelerates pathogen clearance. Therefore, it is possible that IgA not only prevents *C. albicans* adhesion and epithelial invasion it also traps the fungus resulting in enchained growth and clearance.

Reduced salivary flow rate in oral diseased states such as Sjögren's syndrome, or during cytotoxic and radiation therapy increases oral carriage of *Candida* spp. and is associated with an increase in OPC (64, 65). The saliva, as part of the humoral immune system, contains many molecular factors which restrict microbial growth, including antimicrobial peptides and IgA (66, 67). Plasma cells reside in the salivary glands and produce IgA which is then secreted in the saliva (45). Here we show that oral fungal persistence not only increases the migration of IgA^+^ B lymphocytes to sites of *Candida* colonization, fungal persistence also increases IgA amounts in the saliva thus providing a barrier against invading fungi.

Early studies indicated that among patients with CMC over 50% appear to have reduced IgA antibodies (68). The most common humoral immune immunodeficiency is inherited selective IgA deficiency (69). Although selective IgA deficiency is a mild form of immunodeficiency, some patients develop a variety of significant clinical problems, such as CMC (70). Furthermore, *Candida* infections in individuals with X-linked agammaglobulinemia (XLA), a mutation in the gene encoding for Bruton tyrosine kinase (BTK) which leads to impaired peripheral B cell maturation, have been described (71–73). Lymphoid cancers patients treated with ibrutinib, a BTK inhibitor, develop invasive fungal infections including candidemia (74). In this context, patients targeted by B cell therapy using the anti-CD20 monoclonal antibody rituximab present with candidemia (75). Considering that systemic *Candida* infections predominantly originate from mucosal barriers (76, 77) B cell responses therefore limit, in part, commensal fungal proliferation and dissemination from mucosal sites. β-glucan, a fungal cell wall component, is able to directly activate B lymphocytes leading to a proinflammatory cytokine response (78), however this mechanism seems dispensable during acute OPC since mice with B cell deficiency are not more susceptibility to pathogenic *C. albicans* infection (79–81). Because mice lacking B cells exhibited an increase in oral commensal *C. albicans* carriage without causing severe disease we propose that tissue-resident B lymphocytes, in conjunction with IgA, maintain a stable commensal fungal community in the oral cavity, while T cells in particular Th17 cells prevent commensal breakthrough and severe disease. Thus, mucosal B lymphocytes and their antibody responses monitor fungal exposure in the oral cavity to sustain commensal tolerance and immunity.

The finding that *C. albicans* oral colonization increases the IgA production leading to increased IgA binding of *S. oralis ex vivo* suggests that fungal colonization may shape the oral microbial community by inducing B cell expansion and cross-specific antibody secretion. It will be of great interest to analyze the contribution of commensal *C. albicans* colonization to oral diseases, such as periodontitis or oral lichen planus.

## Data Availability Statement

The datasets generated for this study can be found in online repositories. The names of the repository/repositories and accession number(s) can be found in the article/Supplementary Material.

## Ethics Statement

The animal study was reviewed and approved by Institutional Animal Care and Use Committee (IACUC) of the Lundquist Institute at Harbor-UCLA Medical Center.

## Author Contributions

MS designed the experiments. MS, NM, and NS performed the experiments and analyzed the data. MS wrote the paper. All authors contributed to the article and approved the submitted version.

## Conflict of Interest

The authors declare that the research was conducted in the absence of any commercial or financial relationships that could be construed as a potential conflict of interest.

## Supplementary Material

Supplementary Table 1 Different expressed genes during fungal commensalism. Differential expression analysis was performed using the DESeq2 R package. The resulting *P*-values were adjusted using the Benjamini and Hochberg's approach for controlling the False Discovery Rate (FDR). Genes with an adjusted *P*-value < 0.05 found by DESeq2 were assigned as differentially expressed genes that showed ratios log_2_ ≥± 0.58 were considered to be different regulated.

Supplementary Figure 1 Gating of infiltrating B lymphocytes. Cells were gated on singlets live B220^+^ CD4^−^ CD8^−^ CD11b^−^ Gr-1^−^ TER-119^−^ EpCam^−^ and distinguished by CD19 and CD138 expression. Plasma cells (PC; CD19^−^ CD138^+^), plasmablasts (PB; CD19^+^ CD138^+^), and B cells (CD19^+^ CD138^−^) were determined.

Supplementary Figure 2 Tissue B220+ antibody depletion. Flow plot of B220^+^ cells in the tongue 11 days post infection commensal infection.

## References

[1] Brown GD Denning DW Gow NA Levitz SM Netea MG White TC. Hidden killers: human fungal infections. Sci Transl Med. (2012) 4:3004404. 10.1126/scitranslmed.3004404 23253612

[2] Benedict K Jackson BR Chiller T Beer KD. Estimation of direct healthcare costs of fungal diseases in the United States. Clin Infect Dis. (2018) 68:1791–7. 10.1093/cid/ciy776 30204844 PMC6409199

[3] Bandara HMHN Panduwawala CP Samaranayake LP. Biodiversity of the human oral mycobiome in health and disease. Oral Dis. (2019) 25:363–71. 10.1111/odi.12899 29786923

[4] Swidergall M Filler SG. Oropharyngeal Candidiasis: fungal invasion and epithelial cell responses. PLoS Pathog. (2017) 13:e1006056. 10.1371/journal.ppat.1006056 28081240 PMC5230744

[5] Verma A Gaffen SL Swidergall M. Innate immunity to mucosal Candida infections. J Fungi. (2017) 3:60. 10.3390/jof3040060 29371576 PMC5753162

[6] Underhill DM Pearlman E. Immune interactions with pathogenic and commensal fungi: a two-way street. Immunity. (2015) 43:845–58. 10.1016/j.immuni.2015.10.023 26588778 PMC4865256

[7] Wheeler ML Limon JJ Bar AS Leal CA Gargus M Tang J. Immunological consequences of intestinal fungal dysbiosis. Cell Host Microbe. (2016) 19:865–73. 10.1016/j.chom.2016.05.003 27237365 PMC4900921

[8] Iliev ID Leonardi I. Fungal dysbiosis: immunity and interactions at mucosal barriers. Nat Rev. Immunol. (2017) 17:635–46. 10.1038/nri.2017.55 28604735 PMC5724762

[9] Miller AW Orr T Dearing D Monga M. Loss of function dysbiosis associated with antibiotics and high fat, high sugar diet. ISME J. (2019) 13:1379–90. 10.1038/s41396-019-0357-4 30700790 PMC6776053

[10] Wilkins LJ Monga M Miller AW. Defining dysbiosis for a cluster of chronic diseases. Sci Rep. (2019) 9:12918. 10.1038/s41598-019-49452-y 31501492 PMC6733864

[11] Richardson JP Moyes DL. Adaptive immune responses to *Candida albicans* infection. Virulence. (2015) 6:327–37. 10.1080/21505594.2015.1004977 25607781 PMC4601188

[12] Jabra-Rizk MA Kong EF Tsui C Nguyen MH Clancy CJ Fidel PL Jr. *Candida albicans* pathogenesis: fitting within the host-microbe damage response framework. Infect Immun. (2016) 84:2724–39. 10.1128/IAI.00469-16 27430274 PMC5038058

[13] Pirofski L-A Casadevall A. Rethinking T cell immunity in oropharyngeal candidiasis. J Exp Med. (2009) 206:269–73. 10.1084/jem.20090093 19204107 PMC2646576

[14] Wang L Zhu L Qin S. Gut microbiota modulation on intestinal mucosal adaptive immunity. J Immunol Res. (2019) 2019:4735040. 10.1155/2019/4735040 31687412 PMC6794961

[15] Hernandez-Santos N Huppler AR Peterson AC Khader SA Mckenna KC Gaffen SL. Th17 cells confer long-term adaptive immunity to oral mucosal *Candida albicans* infections. Mucosal Immunol. (2013) 6:900–10. 10.1038/mi.2012.128 23250275 PMC3608691

[16] Bishu S Hernandez-Santos N Simpson-Abelson MR Huppler AR Conti HR Ghilardi N. The adaptor CARD9 is required for adaptive but not innate immunity to oral mucosal *Candida albicans* infections. Infect Immun. (2014) 82:1173–80. 10.1128/IAI.01335-13 24379290 PMC3958019

[17] Braunsdorf C Leibundgut-Landmann S. Modulation of the fungal-host interaction by the intra-species diversity of *C. albicans. Pathogens*. (2018) 7:11. 10.3390/pathogens7010011 29342100 PMC5874737

[18] Schonherr FA Sparber F Kirchner FR Guiducci E Trautwein-Weidner K Gladiator A. The intraspecies diversity of *C. albicans* triggers qualitatively and temporally distinct host responses that determine the balance between commensalism and pathogenicity. Mucosal Immunol. (2017) 8:2. 10.1038/mi.2017.2 28176789

[19] Kirchner FR Littringer K Altmeier S Tran VDT Schönherr F Lemberg C. Persistence of *Candida albicans* in the oral mucosa induces a curbed inflammatory host response that is independent of immunosuppression. Front Immunol. (2019) 10:330. 10.3389/fimmu.2019.00330 30873177 PMC6400982

[20] Kirchner FR Leibundgut-Landmann S. Tissue-resident memory Th17 cells maintain stable fungal commensalism in the oral mucosa. Mucosal Immunol. (2020). 10.1038/s41385-020-0327-1 32719409 PMC7946631

[21] Macpherson AJ. IgA adaptation to the presence of commensal bacteria in the intestine. Curr Top Microbiol Immunol. (2006) 308:117–36. 10.1007/3-540-30657-9_5 16922088

[22] Macpherson AJ Geuking MB Mccoy KD. Immunoglobulin A: a bridge between innate and adaptive immunity. Curr Opin Gastroenterol. (2011) 27:529–33. 10.1097/MOG.0b013e32834bb805 21912248

[23] Mantis NJ Rol N Corthésy B. Secretory IgA's complex roles in immunity and mucosal homeostasis in the gut. Mucosal Immunol. (2011) 4:603–11. 10.1038/mi.2011.41 21975936 PMC3774538

[24] Macpherson AJ Koller Y Mccoy KD. The bilateral responsiveness between intestinal microbes and IgA. Trends Immunol. (2015) 36:460–70. 10.1016/j.it.2015.06.006 26169256

[25] Li Y Jin L Chen T. The effects of secretory IgA in the mucosal immune system. Biomed Res Int. (2020) 2020:2032057. 10.1155/2020/2032057 31998782 PMC6970489

[26] Corthesy B. Multi-faceted functions of secretory IgA at mucosal surfaces. Front Immunol. (2013) 4:185. 10.3389/fimmu.2013.00185 23874333 PMC3709412

[27] Fonzi WA Irwin MY. Isogenic strain construction and gene mapping in *Candida albicans*. Genetics. (1993) 134:717–28. 8349105 10.1093/genetics/134.3.717 PMC1205510

[28] Rahman D Mistry M Thavaraj S Challacombe SJ Naglik JR. Murine model of concurrent oral and vaginal *Candida albicans* colonization to study epithelial host-pathogen interactions. Microbes Infect. (2007) 9:615–22. 10.1016/j.micinf.2007.01.012 17383212 PMC3242973

[29] Swidergall M Khalaji M Solis NV Moyes DL Drummond RA Hube B. Candidalysin is required for neutrophil recruitment and virulence during systemic *Candida albicans* infection. J Infect Dis. (2019) 220:1477–88. 10.1093/infdis/jiz322 31401652 PMC6761979

[30] Dickson MA Hahn WC Ino Y Ronfard V Wu JY Weinberg RA. Human keratinocytes that express hTERT and also bypass a p16(INK4a)-enforced mechanism that limits life span become immortal yet retain normal growth and differentiation characteristics. Mol Cell Biol. (2000) 20:1436–47. 10.1128/MCB.20.4.1436-1447.2000 10648628 PMC85304

[31] Solis NV Park YN Swidergall M Daniels KJ Filler SG Soll DR. *Candida albicans* white-opaque switching influences virulence but not mating during Oropharyngeal Candidiasis. Infect Immun. (2018) 86:e00774-17. 10.1128/IAI.00774-17 29581190 PMC5964503

[32] Conti HR Bruno VM Childs EE Daugherty S Hunter JP Mengesha BG. IL-17 receptor signaling in oral epithelial cells is critical for protection against Oropharyngeal Candidiasis. Cell Host Microbe. (2016) 20:606–17. 10.1016/j.chom.2016.10.001 27923704 PMC5147498

[33] Altmeier S Toska A Sparber F Teijeira A Halin C Leibundgut-Landmann S. IL-1 coordinates the neutrophil response to *C. albicans* in the oral mucosa. PLoS Pathog. (2016) 12:e1005882. 10.1371/journal.ppat.1005882 27632536 PMC5025078

[34] Solis NV Filler SG. Mouse model of oropharyngeal candidiasis. Nat Protoc. (2012) 7:637–42. 10.1038/nprot.2012.011 22402633 PMC3671943

[35] Solis NV Swidergall M Bruno VM Gaffen SL Filler SG. The aryl hydrocarbon receptor governs epithelial cell invasion during oropharyngeal candidiasis. MBio. (2017) 8:00025–00017. 10.1128/mBio.00025-17 28325761 PMC5362030

[36] Mortazavi A Williams BA Mccue K Schaeffer L Wold B. Mapping and quantifying mammalian transcriptomes by RNA-Seq. Nat Methods. (2008) 5:621–8. 10.1038/nmeth.1226 18516045 PMC13303166

[37] Forche A Solis NV Swidergall M Thomas R Guyer A Beach A. Selection of *Candida albicans* trisomy during oropharyngeal infection results in a commensal-like phenotype. PLoS Genet. (2019) 15:e1008137. 10.1371/journal.pgen.1008137 31091232 PMC6538192

[38] Swidergall M Solis NV Lionakis MS Filler SG EphA2 is an epithelial cell pattern recognition receptor for fungal beta-glucans. Nat Microbiol. (2018) 3:53–61. 10.1038/s41564-017-0059-5 29133884 PMC5736406

[39] Sparber F Leibundgut-Landmann S. Assessment of immune responses to fungal infections: identification and characterization of immune cells in the infected tissue. Methods Mol Biol. (2017) 1508:167–82. 10.1007/978-1-4939-6515-1_8 27837503

[40] Swidergall M Solis NV Wang Z Phan QT Marshall ME Lionakis MS. EphA2 is a neutrophil receptor for *Candida albicans* that stimulates antifungal activity during oropharyngeal infection. Cell Rep. (2019) 28:423–33.e425. 10.1016/j.celrep.2019.06.020 31291578 PMC6638578

[41] Cuomo CA Fanning S Gujja S Zeng Q Naglik JR Filler SG. Genome sequence for *Candida albicans* clinical oral isolate 529L. Microbiol Resour Announc. (2019) 8:e00554–e00519. 10.1128/MRA.00554-19 31221654 PMC6588375

[42] San Millan R Elguezabal N Regulez P Moragues MAD Quindos G Ponton J. Effect of salivary secretory IgA on the adhesion of *Candida albicans* to polystyrene. Microbiology. (2000) 146 (Pt 9):2105–12. 10.1099/00221287-146-9-2105 10974098

[43] Swidergall M. *Candida albicans* at host barrier sites: pattern recognition receptors and beyond. Pathogens. (2019) 8:40. 10.3390/pathogens8010040 30934602 PMC6471378

[44] Ben-Aryeh H Naon H Szargel R Horowitz G Gutman D. The concentration of salivary IgA in whole and parotid saliva and the effect of stimulation. Int J Oral Maxillofac Surg. (1986) 15:81–4. 10.1016/S0300-9785(86)80014-X 3083009

[45] Brandtzaeg P. Secretory IgA: designed for anti-microbial defense. Front Immunol. (2013) 4:222. 10.3389/fimmu.2013.00222 23964273 PMC3734371

[46] Mak TW Saunders ME 20 - Mucosal and Cutaneous Immunity. In: Mak TW Saunders ME, editors. The Immune Response. Burlington, VT: Academic Press (2006). p. 583–609. 10.1016/B978-012088451-3.50022-3

[47] Pabst O Slack E. IgA and the intestinal microbiota: the importance of being specific. Mucosal Immunol. (2020) 13:12–21. 10.1038/s41385-019-0227-4 31740744 PMC6914667

[48] Cole MF Bryan S Evans MK Pearce CL Sheridan MJ Sura PA. Humoral immunity to commensal oral bacteria in human infants: salivary secretory immunoglobulin A antibodies reactive with Streptococcus mitis biovar 1, Streptococcus oralis, Streptococcus mutans, and Enterococcus faecalis during the first two years of life. Infect Immun. (1999) 67:1878–86. 10.1128/.67.4.1878-1886.1999 10085031 PMC96541

[49] Egbuniwe IU Karagiannis SN Nestle FO Lacy KE. Revisiting the role of B cells in skin immune surveillance. Trends Immunol. (2015) 36:102–11. 10.1016/j.it.2014.12.006 25616715

[50] Kato A Hulse KE Tan BK Schleimer RP. B-lymphocyte lineage cells and the respiratory system. J Allergy Clin Immunol. (2013) 131:933–58. 10.1016/j.jaci.2013.02.023 23540615 PMC3628816

[51] Kitamura D Roes J Kühn R Rajewsky K. A B cell-deficient mouse by targeted disruption of the membrane exon of the immunoglobulin mu chain gene. Nature. (1991) 350:423–6. 10.1038/350423a0 1901381

[52] Ghosh S Hoselton SA Schuh JM. μ-chain-deficient mice possess B-1 cells and produce IgG and IgE, but not IgA, following systemic sensitization and inhalational challenge in a fungal asthma model. J Immunol. (2012) 189:1322–9. 10.4049/jimmunol.1200138 22732592 PMC3401271

[53] Puel A Cypowyj S Marodi L Abel L Picard C Casanova JL. Inborn errors of human IL-17 immunity underlie chronic mucocutaneous candidiasis. Curr Opin Allergy Clin Immunol. (2012) 12:616–22. 10.1097/ACI.0b013e328358cc0b 23026768 PMC3538358

[54] Lionakis MS Iliev ID Hohl TM Immunity against fungi. JCI Insight. (2017) 2:93156 10.1172/jci.insight.93156 28570272 PMC5453709

[55] Li J Casanova JL Puel A. Mucocutaneous IL-17 immunity in mice and humans: host defense vs. excessive inflammation Mucosal Immunol. (2018) 11:581–9. 10.1038/mi.2017.97 29186107 PMC5975098

[56] Bar E Gladiator A Bastidas S Roschitzki B Acha-Orbea H Oxenius A. A novel Th cell epitope of *Candida albicans* mediates protection from fungal infection. J Immunol. (2012) 188:5636–43. 10.4049/jimmunol.1200594 22529294

[57] Trautwein-Weidner K Gladiator A Kirchner FR Becattini S Rülicke T Sallusto F. Antigen-specific Th17 cells are primed by distinct and complementary dendritic cell subsets in oropharyngeal candidiasis. PLoS Pathog. (2015) 11:e1005164. 10.1371/journal.ppat.1005164 26431538 PMC4591991

[58] Panda S Ding JL. Natural antibodies bridge innate and adaptive immunity. J Immunol. (2015) 194:13–20. 10.4049/jimmunol.1400844 25527792

[59] Mathias A Pais B Favre L Benyacoub J Corthésy B. Role of secretory IgA in the mucosal sensing of commensal bacteria. Gut Microbes. (2014) 5:688–95. 10.4161/19490976.2014.983763 25536286 PMC4615909

[60] Williams DW Potts AJC Wilson MJ Matthews JB Lewis MAO. Characterisation of the inflammatory cell infiltrate in chronic hyperplastic candidosis of the oral mucosa. J Oral Pathol Med. (1997) 26:83–9. 10.1111/j.1600-0714.1997.tb00026.x 9049907

[61] Williams A Williams D Rogers H Wei X Lewis M Wozniak S. Immunohistochemical expression patterns of inflammatory cells involved in chronic hyperplastic candidosis. Pathogens. (2019) 8:232. 10.3390/pathogens8040232 31718115 PMC6963680

[62] Ho J Yang X Nikou SA Kichik N Donkin A Ponde NO. Candidalysin activates innate epithelial immune responses via epidermal growth factor receptor. Nat Commun. (2019) 10:2297. 10.1038/s41467-019-09915-2 31127085 PMC6534540

[63] Moor K Diard M Sellin ME Felmy B Wotzka SY Toska A. High-avidity IgA protects the intestine by enchaining growing bacteria. Nature. (2017) 544:498–502. 10.1038/nature22058 28405025

[64] Hernandez YL Daniels TE. Oral candidiasis in Sjogren's syndrome: prevalence, clinical correlations, and treatment. Oral Surg Oral Med Oral Pathol. (1989) 68:324–9. 10.1016/0030-4220(89)90218-1 2788854

[65] Nadig SD Ashwathappa DT Manjunath M Krishna S Annaji AG Shivaprakash PK. A relationship between salivary flow rates and Candida counts in patients with xerostomia. J Oral Maxillofac Pathol. (2017) 21:316. 10.4103/jomfp.JOMFP_231_16 28932047 PMC5596688

[66] Brandtzaeg P. Secretory immunity with special reference to the oral cavity. J Oral Microbiol. (2013) 5. 10.3402/jom.v5i0.20401 23487566 PMC3595421

[67] Swidergall M Ernst JF. Interplay between Candida albicans and the antimicrobial peptide armory. Eukaryot Cell. (2014) 13:950–7. 10.1128/EC.00093-14 24951441 PMC4135787

[68] Lehner T Wilton JM Ivanyi L. Immunodeficiencies in chronic muco-cutaneous candidosis. Immunology. (1972) 22:775–87. 4336639 PMC1407855

[69] Odineal DD Gershwin ME. The epidemiology and clinical manifestations of autoimmunity in selective IgA deficiency. Clin Rev Allergy Immunol. (2020) 58:107–33. 10.1007/s12016-019-08756-7 31267472

[70] Kalfa VC Roberts RL Stiehm ER. The syndrome of chronic mucocutaneous candidiasis with selective antibody deficiency. Ann Allergy Asthma Immunol. (2003) 90:259–64. 10.1016/S1081-1206(10)62152-7 12602677

[71] Mamishi S Eghbali AN Rezaei N Abolhassani H Parvaneh N Aghamohammadi A. A single center 14 years study of infectious complications leading to hospitalization of patients with primary antibody deficiencies. Braz J Infect Dis. (2010) 14:351–5. 10.1016/S1413-8670(10)70074-X 20963319

[72] Preece K Lear G. X-linked agammaglobulinemia with normal immunoglobulin and near-normal vaccine seroconversion. Pediatrics. (2015) 136:e1621–4. 10.1542/peds.2014-3907 26527549

[73] Xu Y Qing Q Liu X Chen S Chen Z Niu X. Bruton's agammaglobulinemia in an adult male due to a novel mutation: a case report. J Thorac Dis. (2016) 8:E1207–12. 10.21037/jtd.2016.10.12 27867589 PMC5107543

[74] Varughese T Taur Y Cohen N Palomba ML Seo SK Hohl TM. Serious infections in patients receiving ibrutinib for treatment of lymphoid cancer. Clin Infect Dis. (2018) 67:687–92. 10.1093/cid/ciy175 29509845 PMC6093991

[75] Van Vollenhoven RF Emery P Bingham CO III Keystone EC Fleischmann RM Furst DE. Long-term safety of rituximab in rheumatoid arthritis: 9.5-year follow-up of the global clinical trial programme with a focus on adverse events of interest in RA patients. Ann Rheum Dis. (2013) 72:1496–502. 10.1136/annrheumdis-2012-201956 23136242 PMC3756452

[76] Nucci M Anaissie E Revisiting the source of candidemia: skin or gut? Clin Infect Dis. (2001) 33:1959–67. 10.1086/323759 11702290

[77] Pappas PG Lionakis MS Arendrup MC Ostrosky-Zeichner L Kullberg BJ Invasive candidiasis. Nat Rev Dis Primers. (2018) 4:18026 10.1038/nrdp.2018.26 29749387

[78] Ali MF Driscoll CB Walters PR Limper AH Carmona EM. β-glucan-activated human B lymphocytes participate in innate immune responses by releasing proinflammatory cytokines and stimulating neutrophil chemotaxis. J Immunol. (2015) 195:5318–26. 10.4049/jimmunol.1500559 26519534 PMC4655155

[79] Carrow EW Hector RF Domer JE. Immunodeficient CBA/N mice respond effectively to *Candida albicans*. Clin Immunol Immunopathol. (1984) 33:371–80. 10.1016/0090-1229(84)90308-8 6388927

[80] Jensen J Warner T Balish E Resistance of SCID mice to *Candida albicans* administered intravenously or colonizing the gut: role of polymorphonuclear leukocytes and macrophages. J Infect Dis. (1993) 167:912–9. 10.1093/infdis/167.4.912 8383723

[81] Jensen J Warner T Balish E. The role of phagocytic cells in resistance to disseminated candidiasis in granulocytopenic mice. J Infect Dis. (1994) 170:900–5. 10.1093/infdis/170.4.900 7930734

