# Supplementary figures and images for: Mucosal IgA Prevents Commensal Candida albicans Dysbiosis in the Oral Cavity
Source: Front Immunol. 2020 Oct 22;11:555363. doi: 10.3389/fimmu.2020.555363 (PMC7642201; doi:10.3389/fimmu.2020.555363)

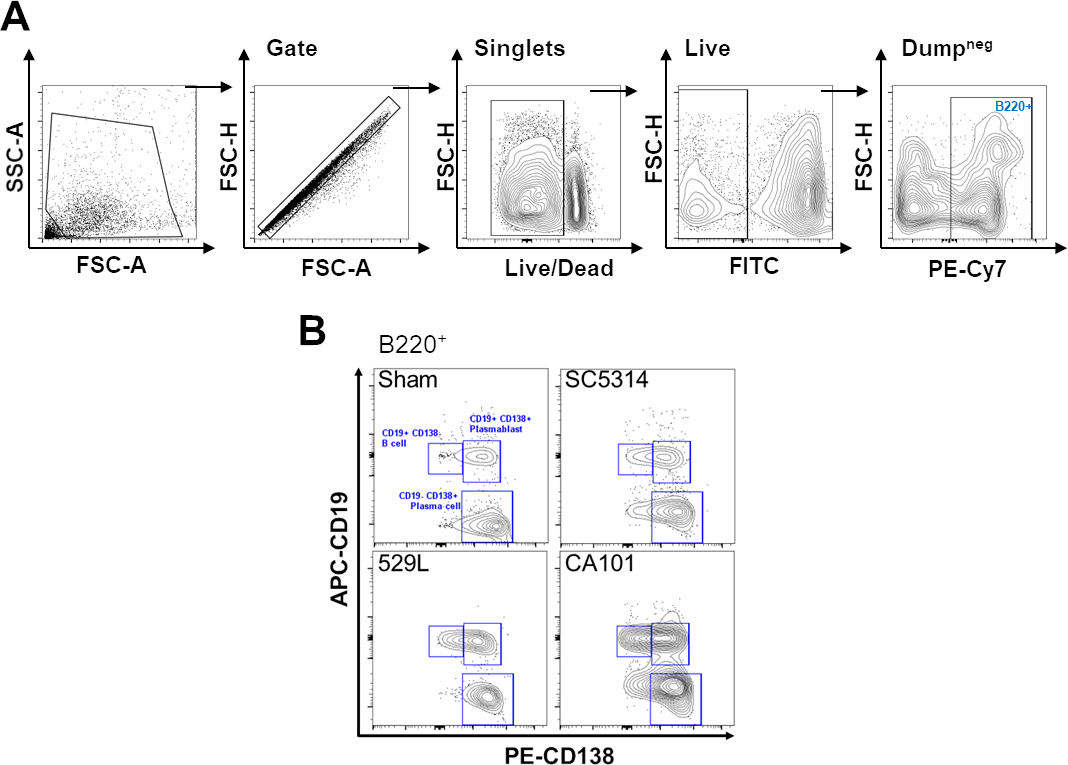

Supplement: Supplementary Figure 1 — Gating of infiltrating B lymphocytes. Cells were gated on singlets live B220+ CD4− CD8− CD11b− Gr-1− TER-119− EpCam− and distinguished by CD19 and CD138 expression. Plasma cells (PC; CD19− CD138+), plasmablasts (PB; CD19+ CD138+), and B cells (CD19+ CD138−) were determined. [file Image_1.TIF]

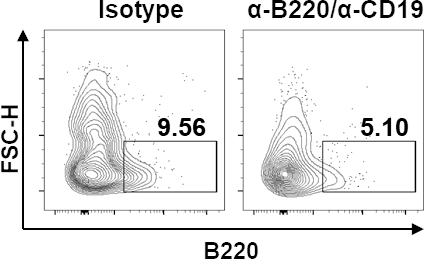

Supplement: Supplementary Figure 2 — Tissue B220+ antibody depletion. Flow plot of B220+ cells in the tongue 11 days post infection commensal infection. [file Image_2.TIF]
